# Delayed local recurrence of pancreatic adenosquamous cell carcinoma after curative surgery: A case report

**DOI:** 10.1016/j.ijscr.2022.107735

**Published:** 2022-10-11

**Authors:** Atsuhiro Watanabe, Tomoyuki Abe, Akihiko Oshita, Keiji Hanada, Toshio Noriyuki, Masahiro Nakahara

**Affiliations:** a Department of Surgery, Onomichi General Hospital, 1-10-23 Hirahara, Onomichi, Hiroshima 722-8508, Japan; b Department of Gastroenterology, Onomichi General Hospital, 1-10-23 Hirahara, Onomichi, Hiroshima 722-8508, Japan; c Department of Surgery, National Hospital Organization Higashihiroshima Medical Center, 513 Jike, Saijo, Higashihiroshima, Hiroshima 739-0041, Japan

**Keywords:** CEA, carcinoembryonic antigen, EUS, endoscopic ultrasonography, FNA, fine needle aspiration, MRI, magnetic resonance imaging, OS, overall survival, PASC, pancreatic adenosquamous cell carcinoma, PDAC, pancreatic ductal adenocarcinoma, PET, positron emission tomography, SCC, squamous cell carcinoma, Pancreatic cancer, Adenosquamous cell carcinoma, Distal pancreatectomy

## Abstract

**Introduction:**

Pancreatic adenosquamous cell carcinoma (PASC) is a rare histological type of pancreatic malignancy with a particularly poor prognosis, even after curative surgery. Here, we describe the long-term prognosis of PASC in a patient who developed delayed local recurrence of the remnant pancreas after successful distal pancreatectomy, together with a literature review.

**Presentation of case:**

A 59-year-old woman had a history of hepatitis C. Computed tomography revealed a hypointense mass in the pancreatic body in the arterial phase of the study. Magnetic resonance imaging revealed a tumor (20 mm) in the pancreatic body and dilatation of the main pancreatic duct at the periphery of the tumor. The patient was diagnosed with resectable pancreatic ductal adenocarcinoma and underwent distal pancreatectomy with lymphadenectomy; her postoperative course was uneventful. Immunohistochemical analysis of the resected specimen confirmed the diagnosis of tumor node metastasis [TNM] classification T2N1M0 stage IIB. Five years after curative surgery, following adjuvant systemic chemotherapy with S-1, local recurrence in the remnant pancreas occurred, which invaded the common hepatic artery and celiac pleural plexus. Systemic chemotherapy with gemcitabine and abraxiane is currently underway.

**Discussion:**

Curative surgery significantly affects the prognosis of patients with PASC. Adjuvant chemotherapy may prolong the survival of these patients. Delayed remnant pancreatic recurrence should be considered during the surveillance of pancreatic cancer after curative resection.

**Conclusion:**

We present a case of PASC in a patient who developed local recurrence in the remnant pancreas 5 years after successful distal pancreatectomy. Special attention should be paid not only to early recurrence but also to delayed local recurrence in PASC.

## Highlights

• Pancreatic adenosquamous cell carcinoma (PASC) is a rare histological type of pancreatic malignancy
• We describe the long-term prognosis of PASC in a patient who developed delayed local recurrence of the remnant pancreas after distal pancreatectomy.
• Delayed remnant pancreatic recurrence should be considered during the surveillance of pancreatic cancer after curative resection.

## 1 Introduction

Pancreatic cancer has one of the lowest 5-year relative survival rates among all gastrointestinal cancers [1]. Pancreatic ductal adenocarcinoma (PDAC) is the most common type of pancreatic cancer, accounting for >90 % of all pancreatic malignancies [2], [3], [4], [5]. Approximately 40,000 patients were newly diagnosed with pancreatic cancer, and 33,000 patients died of this malignancy in Japan [6]. The 5-year overall survival (OS) rate is 12.4 % for locally advanced pancreatic cancer and only 1.8 % for metastatic pancreatic cancer [7].

Pancreatic adenosquamous cell carcinoma (PASC) is a rare histological type of pancreatic malignancy, which accounts for 1–4 % of all pancreatic cancers, and has a particularly poor prognosis [2], [5], [8]. Surgical resection remains the treatment of choice even for PASC; however, the 2-year survival rate among patients with PASC with R0 resection is approximately 30 %, and the median survival is only 11–18 months [3], [5]. Early distant recurrence in the lung and liver results in a dismal prognosis of PASC rather than ordinary PDAC.

Herein, we report the case of a patient with PASC who developed local recurrence in the remnant pancreas over 5 years after radical pancreatectomy with postoperative adjuvant chemotherapy.

This study was reported in accordance with the SCARE 2020 criteria [9].

## 2 Presentation of case

**Fig. 1:**
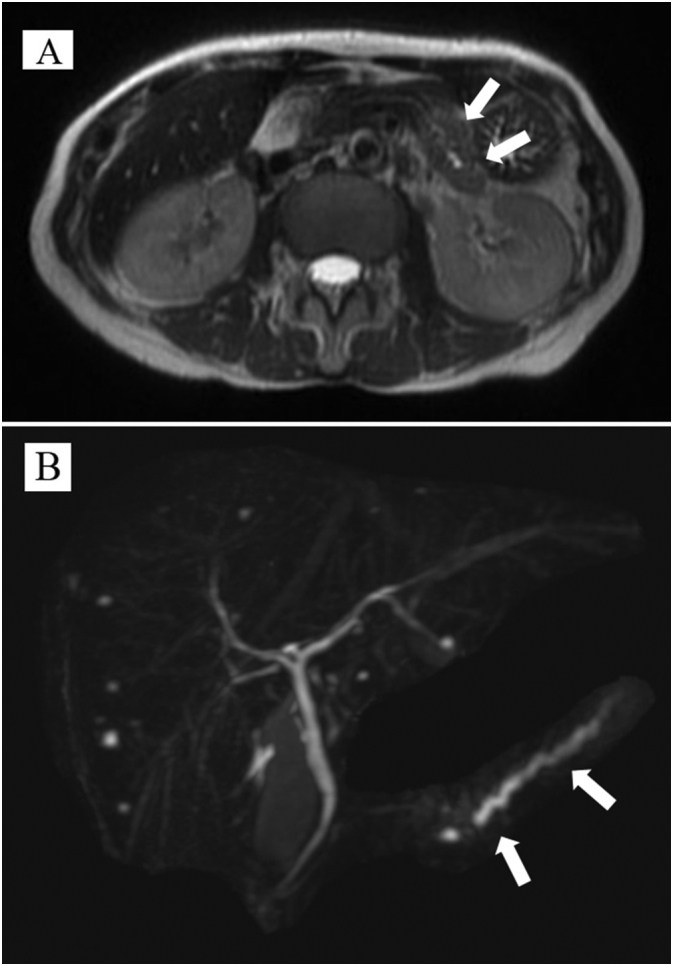
MRI scan findings. A: A tumor showing faint, high signal intensity (white arrow) on a T2-weighted image. B: The main pancreatic duct is dilated (white arrow) secondary to the tumor obstruction. MRI: magnetic resonance imaging.

**Fig. 2:**
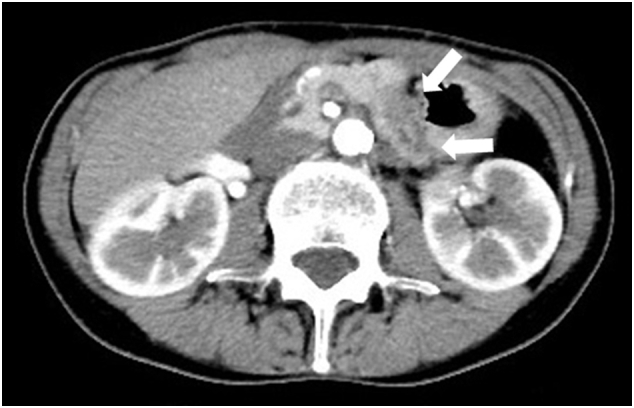
Abdominal dynamic CT scan findings. A: A low-density mass is observed (white arrow) during the arterial phase of the study. CT: computed tomography.

**Fig. 3:**
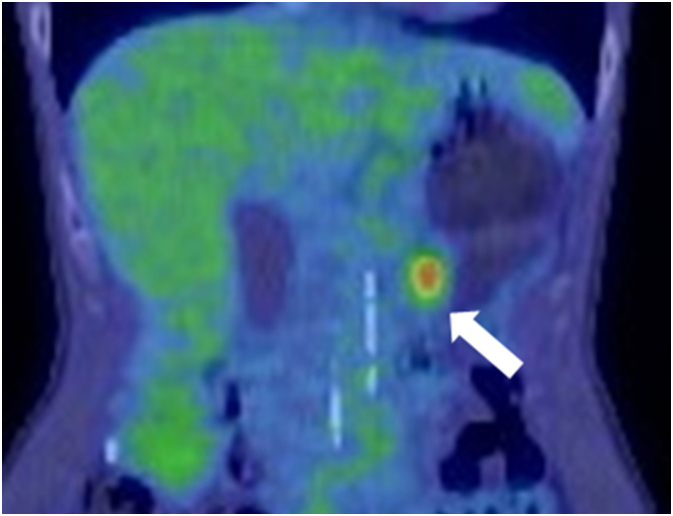
PET-CT scan findings. Abnormal accumulation of tracer is observed (white arrow) with significantly high SUVmax of 4.2. PET-CT, positron emission tomography computed tomography; SUVmax, maximum standardized uptake value.

**Fig. 4:**
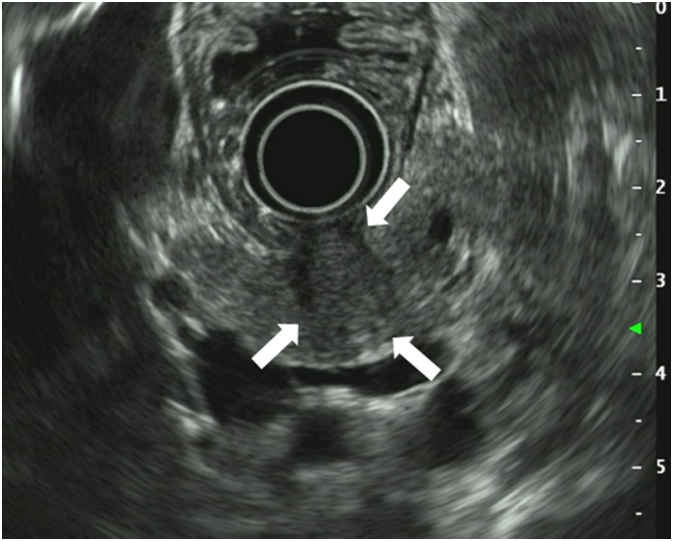
EUS scan findings. The tumor is visualized as a hypoechoic mass (white arrow) that measures 19 mm in diameter. EUS: endoscopic ultrasonography.

A 59-year-old asymptomatic woman was diagnosed with a tumor (20 mm) in the pancreatic body based on magnetic resonance imaging (MRI) findings during annual follow-up for hepatitis C. She had no family history of cancer, no history of smoking, but drank occasionally. Blood test results for tumor markers were within the normal range (carcinoembryonic antigen (CEA), 0.7 ng/mL; carbohydrate antigen 19-9, 24.3 U/mL; and the Duke pancreatic monoclonal antigen type 2, 25 U/mL). T2-weighted MRI showed a pancreatic body tumor with high signal intensity. Diffusion-weighted images also revealed a neoplasm with high signal intensity, and main pancreatic duct dilatation was detected at the tumor periphery (Fig. 1). Computed tomography (CT) revealed a hypointense mass with an indistinct border in the pancreatic body during the arterial phase (Fig. 2) with persistent low enhancement during the delayed phase of the study. Positron emission tomography revealed abnormal uptake by the pancreatic tumor (maximum standardized uptake value, 4.2) (Fig. 3). Endoscopic ultrasonography (EUS) revealed a hypoechoic mass (maximum size, 19 mm) in the distal portion of the pancreas with obstruction of the main pancreatic duct and dilatation of the caudal pancreatic duct (Fig. 4). Pancreatic ductography revealed a tumor-induced obstruction of the main pancreatic duct at the level of the pancreatic body. The patient was preoperatively diagnosed with resectable PDAC.

**Fig. 5:**
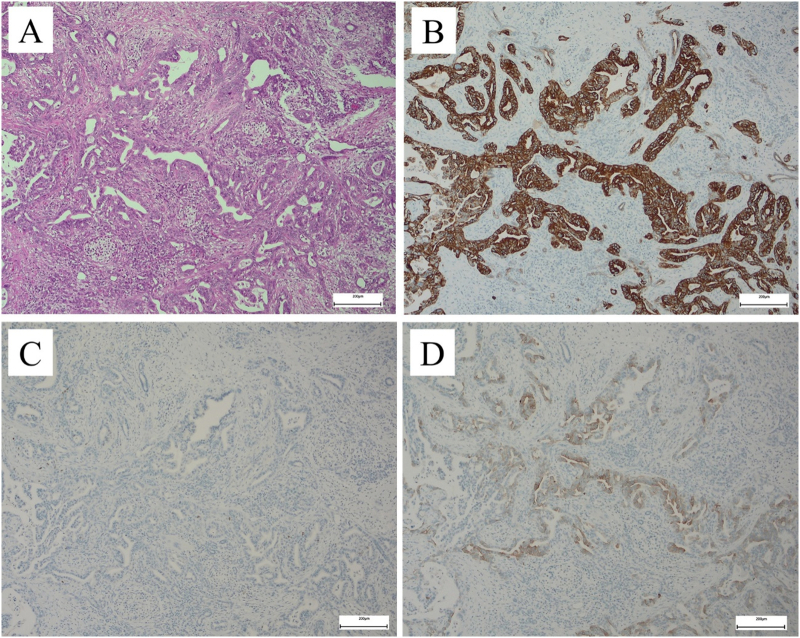
Histopathological findings in the resected specimen. A: The tumor consists of mainly papillary adenocarcinoma and some SCC components with prominent cytoplasmic eosinophilia and nuclear eccentricity (hematoxylin & eosin). B: Immunohistochemical analysis showing positive 34βE12 expression in the SCC and a portion of the adenocarcinoma. C: Cells with immunonegativity for p40. D: Cells showing partial immunopositivity for CK5/6. SCC: squamous cell carcinoma.

We performed an open distal pancreatectomy with lymphadenectomy. The operation time was 222 min, and the estimated blood loss was 20 g. The patient showed a good postoperative course and was discharged on day 12 after surgery. Histopathological examination of the resected tumor showed a combination of squamous cell carcinoma (SCC) and adenocarcinoma (Fig. 5), and the patient was diagnosed with PASC (T2N1M0 Stage IIB). One of the 48 lymph nodes was positive for metastasis with lymphovascular and perineural invasion. We initiated adjuvant systemic chemotherapy with S-1 (80 mg/day, 4-day dosing with 3-day rest) for 1 year, and the patient did not show recurrence for 5 years postoperatively. However, 5 years and 2 months after surgery, the CEA level was elevated at 25.2 ng/mL. EUS revealed a hypoechoic mass (maximum size, 30 mm) in the residual pancreas. EUS-fine needle aspiration (FNA) was performed; unfortunately, the patient was diagnosed with recurrent PDAC in the remnant pancreas, which already invaded the common hepatic artery and pleural plexus on CT. She is currently being treated with nab-paclitaxel plus gemcitabine.

## 3 Discussion

PASC is a rare histological type of pancreatic cancer, accounting for 1 %–4 % of all pancreatic cancers [2], [10], [11]. Usually, PASC shows aggressive behavior, and the prognosis even after curative surgery remains poorer than that of conventional PDAC [2], [10], [11], [12]. Boecker et al. reported that the median OS of patients with PASC tends to be worse than that of PDAC (8.2 months vs. 20.1 months, *P* = 0.089) [10]. Curative surgery significantly affects the prognosis of patients with PASC and that of patients with PDAC [3], [4], [13]. Positive lymph nodes, margin-positive resections, and older age (≥65 years) are associated with poor prognosis in patients with PASC [5], [8], [13]. In our case, regardless of lymph node metastasis, curative resection with postoperative adjuvant systemic chemotherapy using S-1 contributed to the 5-year recurrence-free survival. Delayed remnant pancreatic recurrence occurred; however, considering the different tumor pathology and location, this is not an ordinary remnant pancreatic recurrence. EUS-FNA revealed that the main tumor was adenocarcinoma, not squamous cell carcinoma (SCC).

Perioperative systemic chemotherapy together with pancreatectomy is the standard treatment for resectable and borderline PDAC [4], [5], [8], [12]; however, the optimal perioperative systemic chemotherapy for PASC remains unknown. Wild et al. reported that a platinum agent used as a component of adjuvant therapy prolonged recurrence-free survival in patients with PASC [8]. Notably, patients with PASC at a high risk for recurrence, such as those with a large tumor diameter, advanced T stage, and tumor differentiation, tend to show high sensitivity to adjuvant chemotherapy [5]. Despite the unavailability of a well-established perioperative systemic chemotherapy regimen for patients with PASC, curative surgery following adjuvant chemotherapy may prolong the survival of these patients, as observed in our case. Aigner et al. previously reported on the effectiveness of intra-arterial infusion chemotherapy and isolated upper abdominal perfusion chemotherapy [14]. Further study would be required to evaluate appropriate adjuvant chemotherapy for PASC.

The malignant potential of PASC is strongly associated with the SCC component of the tumor. Adenosquamous cell carcinomas that originate from organs lined by glandular epithelium, such as the colon, rectum, breast, and prostate, are rare, and their clinical prognosis is poorer and their behavior is more aggressive than that of conventional adenocarcinoma [15], [16], [17]. Several studies have reported significantly shorter doubling times for SCC than for lung adenocarcinoma [18], [19], and some studies have reported that PASC tumors are larger than PDAC lesions [3], [4], [5], [10], [13].

The pathogenesis of PASC remains unclear, although the following hypotheses are prevalent: (a) origin from cancer stem cells, (b) squamous metaplasia of the intestinal mucosa, (c) malignant squamous metaplastic transformation of adenocarcinoma, and (d) collision of the two components. The cancer stem cell hypothesis is considered the most likely pathogenetic contributor, as reported in previous studies [2], [20], [21]. However, the association between the number of SCC components and prognosis remains unclear. Voong et al. reported that the percentage of squamous differentiation was not associated with median OS (<30 % vs. ≥30 %) [5]. By contrast, some studies have reported that progression patterns of the SCC component are characterized by vascular invasion as opposed to metastasis to the surrounding lymph nodes [2], [5], [22], which may explain the greater tendency of PASC to show vascular invasion and distant metastasis to the lungs and liver compared with features of PDAC [22], [23]. It is hypothesized that proliferation of the SCC component of PASC results in distant metastasis to the lungs and liver and is therefore associated with a poorer prognosis than that of PDAC.

Up to 80 % of patients who undergo curative pancreatectomy experience systemic or local recurrence within 2 years [24], [25]. Kleeff et al. reported that recurrent pancreatic cancer in the remnant pancreas after initial pancreatectomy in the absence of systemic disease is extremely rare, with an incidence of 3.1 % [26]. Yamada et al. reported a median survival of 26 months in patients with remnant pancreatic recurrence who underwent resection [27]. Considering the long interval from initial pancreatectomy to the diagnosis of remnant pancreatic cancer, it is quite difficult to distinguish metachronous remnant pancreatic recurrence from a second primary pancreatic cancer [27], [28], [29]. We present a case of PASC in a patient who developed remnant pancreatic cancer 5 years after successful distal pancreatectomy. We regard the recurrent tumor as a second primary pancreatic cancer because the histological findings and location of the tumor were different from those of the tumors from the primary operation. In clinical situations, delayed remnant pancreatic recurrence should be considered during the surveillance of pancreatic cancer after curative resection.

## 4 Conclusion

PASC is a rare histological type of pancreatic cancer with a poorer prognosis than conventional PDAC. Special attention should be paid to not only the early recurrence of PASC but also delayed remnant pancreatic cancer.

## Funding

This research did not receive any specific grant from funding agencies in the public, commercial, or not-for-profit sectors.

## Ethical approval

All procedures used in this study were approved by the ethics committee of our institution.

## Consent

Written informed consent was obtained from the patient for publication of this case report and accompanying images. A copy of the written consent is available for review by the Editor-in-Chief of this journal on request.

## Author contribution

AW drafted the manuscript. TA conceived of the idea and developed the theory. TA performed the surgery. SY prepared the histopathological images. All authors have discussed the results and approved the final manuscript.

## Research registration number

Not applicable.

## Guarantor

Tomoyuki Abe.

## Declaration of competing interest

The authors declare that they have no known competing financial interests or personal relationships that could have influenced the work reported in this study.

## References

[1] Siegel R.L., Miller K.D., Jemal A. (2020). Cancer statistics, 2020. CA Cancer J. Clin..

[2] Simone C.G., Zuluaga Toro T., Chan E., Feely M.M., Trevino J.G., George T.J. (2013). Characteristics and outcomes of adenosquamous carcinoma of the pancreas. Gastrointest. Cancer Res..

[3] Boyd C.A., Benarroch-Gampel J., Sheffield K.M., Cooksley C.D., Riall T.S. (2012). 415 patients with adenosquamous carcinoma of the pancreas: a population-based analysis of prognosis and survival. J. Surg. Res..

[4] Katz M.H., Taylor T.H., Al-Refaie W.B., Hanna M.H., Imagawa D.K., Anton-Culver H. (2011). Adenosquamous versus adenocarcinoma of the pancreas: a population-based outcomes analysis. J. Gastrointest. Surg..

[5] Voong K.R., Davison J., Pawlik T.M., Uy M.O., Hsu C.C., Winter J. (2010). Resected pancreatic adenosquamous carcinoma: clinicopathologic review and evaluation of adjuvant chemotherapy and radiation in 38 patients. Hum. Pathol..

[6] Hori M., Matsuda T., Shibata A., Katanoda K., Sobue T., Nishimoto H. (2015). Cancer incidence and incidence rates in Japan in 2009: a study of 32 population-based cancer registries for the Monitoring of Cancer Incidence in Japan (MCIJ) project. Jpn. J. Clin. Oncol..

[7] Matsuda T., Ajiki W., Marugame T., Ioka A., Tsukuma H., Sobue T. (2011). Population-based survival of cancer patients diagnosed between 1993 and 1999 in Japan: a chronological and international comparative study. Jpn. J. Clin. Oncol..

[8] Wild A.T., Dholakia A.S., Fan K.Y., Kumar R., Moningi S., Rosati L.M. (2015). Efficacy of platinum chemotherapy agents in the adjuvant setting for adenosquamous carcinoma of the pancreas. J. Gastrointest. Oncol..

[9] Agha R.A., Borrelli M.R., Farwana R., Koshy K., Fowler A.J., Orgill D.P. (2018). The PROCESS 2018 statement: updating consensus Preferred Reporting Of CasE Series in Surgery (PROCESS) guidelines. Int. J. Surg..

[10] Boecker J., Feyerabend B., Tiemann K., Buchwalow I., Wagner K.C., Oldhafer K.J. (2020). Adenosquamous carcinoma of the pancreas comprise a heterogeneous group of tumors with the worst outcome: a clinicopathological analysis of 25 cases identified in 562 pancreatic carcinomas resected with curative intent. Pancreas.

[11] Imaoka H., Shimizu Y., Mizuno N., Hara K., Hijioka S., Tajika M. (2014). Clinical characteristics of adenosquamous carcinoma of the pancreas: a matched case-control study. Pancreas.

[12] Hue J.J., Katayama E., Sugumar K., Winter J.M., Ammori J.B., Rothermel L.D. (2021). The importance of multimodal therapy in the management of nonmetastatic adenosquamous carcinoma of the pancreas: analysis of treatment sequence and strategy. Surgery.

[13] Hester C.A., Augustine M.M., Choti M.A., Mansour J.C., Minter R.M., Polanco P.M. (2018). Comparative outcomes of adenosquamous carcinoma of the pancreas: an analysis of the National Cancer Database. J. Surg. Oncol..

[14] Aigner K.R., Gailhofer S., Selak E., Aigner K. (2019). Intra-arterial infusion chemotherapy versus isolated upper abdominal perfusion for advanced pancreatic cancer: a retrospective cohort study on 454 patients. J. Cancer Res. Clin. Oncol..

[15] Masoomi H., Ziogas A., Lin B.S., Barleben A., Mills S., Stamos M.J. (2012). Population-based evaluation of adenosquamous carcinoma of the colon and rectum. Dis. Colon Rectum.

[16] Soo K., Tan P.H. (2013). Low-grade adenosquamous carcinoma of the breast. J. Clin. Pathol..

[17] Wang J., Wang F.W., Lagrange C.A., Hemstreet G.P. (2010). Clinical features and outcomes of 25 patients with primary adenosquamous cell carcinoma of the prostate. Rare Tumors.

[18] Honda O., Johkoh T., Sekiguchi J., Tomiyama N., Mihara N., Sumikawa H. (2009). Doubling time of lung cancer determined using three-dimensional volumetric software: comparison of squamous cell carcinoma and adenocarcinoma. Lung Cancer.

[19] Wilson D.O., Ryan A., Fuhrman C., Schuchert M., Shapiro S., Siegfried J.M. (2012). Doubling times and CT screen-detected lung cancers in the Pittsburgh Lung Screening Study. Am. J. Respir. Crit. Care Med..

[20] Fang Y., Su Z., Xie J., Xue R., Ma Q., Li Y. (2017). Genomic signatures of pancreatic adenosquamous carcinoma (PASC). J. Pathol..

[21] Moslim M.A., Lefton M.D., Ross E.A., Mackrides N., Reddy S.S. (2021). Clinical and histological basis of adenosquamous carcinoma of the pancreas: a 30-year experience. J. Surg. Res..

[22] Hoshimoto S., Hoshi N., Hishinuma S., Shirakawa H., Tomikawa M., Ozawa I. (2017). Clinical implications of the proliferative ability of the squamous component regarding tumor progression of adenosquamous carcinoma of the pancreas: a preliminary report. Pancreatology.

[23] Komatsu H., Egawa S., Motoi F., Morikawa T., Sakata N., Naitoh T. (2015). Clinicopathological features and surgical outcomes of adenosquamous carcinoma of the pancreas: a retrospective analysis of patients with resectable stage tumors. Surg. Today.

[24] Garcea G., Dennison A.R., Pattenden C.J., Neal C.P., Sutton C.D., Berry D.P. (2008). Survival following curative resection for pancreatic ductal adenocarcinoma. A systematic review of the literature. JOP.

[25] Smeenk H.G., Tran T.C., Erdmann J., van Eijck C.H., Jeekel J. (2005). Survival after surgical management of pancreatic adenocarcinoma: does curative and radical surgery truly exist?. Langenbeck's Arch. Surg..

[26] Kleeff J., Reiser C., Hinz U., Bachmann J., Debus J., Jaeger D. (2007). Surgery for recurrent pancreatic ductal adenocarcinoma. Ann. Surg..

[27] Yamada S., Kobayashi A., Nakamori S., Baba H., Yamamoto M., Yamaue H. (2018). Resection for recurrent pancreatic cancer in the remnant pancreas after pancreatectomy is clinically promising: results of a project study for pancreatic surgery by the Japanese Society of Hepato-Biliary-Pancreatic Surgery. Surgery.

[28] Choi M., Kim N.W., Hwang H.K., Lee W.J., Kang C.M. (2020). Repeated pancreatectomy for isolated local recurrence in the remnant pancreas following radical pancreatectomy for pancreatic ductal adenocarcinoma: a pooled analysis. J Clin Med.

[29] Hashimoto D., Chikamoto A., Ohmuraya M., Sakata K., Miyake K., Kuroki H. (2014). Pancreatic cancer in the remnant pancreas following primary pancreatic resection. Surg. Today.

